# Impact on cell to plasma ratio of miR-92a in patients with acute leukemia: in vivo assessment of cell to plasma ratio of miR-92a

**DOI:** 10.1186/1756-0500-3-347

**Published:** 2010-12-24

**Authors:** Junko H Ohyashiki, Tomohiro Umezu, Chiaki Kobayashi, Ryoko S Hamamura, Masami Tanaka, Masahiko Kuroda, Kazuma Ohyashiki

**Affiliations:** 1 Institute of Medical Science, Tokyo Medical University, Tokyo, Japan; 2 First Department of Internal Medicine, Tokyo Medical University, Tokyo, Japan; 3 Department of Molecular Pathology, Tokyo Medical University, Tokyo, Japan

## Abstract

**Background:**

Plasma microRNA (miRNA) has become a promising biomarker for detecting cancer; however, it remains uncertain whether miRNA expression levels in plasma reflect those in tumor cells. Our aim was to determine the biological relevance of miR-92a, which has been implicated as an oncomiR in both plasma and leukemia cells in patients with acute leukemia and to evaluate whether it could be a novel biomarker for monitoring these patients.

**Results:**

We quantified the expression level of miR-92a in both cells and plasma by reverse transcription polymerase chain reaction in 91 patients with acute leukemia. We also determined miR-92a expression levels in peripheral blood mononuclear cells (PBMNC) from normal controls. We compared miR-92a expression in plasma with its expression in leukemia cells. Synthetic anti-miR-92a inhibitor was transfected into Raji and OM9;22 cells, and apoptosis was assessed. For in vivo assessment, 6-week-old female nude mice were injected with U937 cells, and miR-92a expression in plasma and tumors was measured. The level of miR-92a expression in fresh leukemia cells was highly variable compared with PBMNC, but significantly lower compared with CD34-positive cells obtained from healthy volunteers. We also noticed that miR-92a was preferentially expressed in acute lymphoblastic leukemia (ALL) cells in comparison with acute myeloid leukemia (AML) cells. More specifically, cellular miR-92a expression was significantly increased in a subset of ALL cells, and ALL patients with overexpressed miR-92a had poor prognoses. The anti-miR-92a inhibitor-treated Raji and OM9;22 cells revealed an increase of apoptotic cells. Notably, the cell to plasma ratio of miR-92a expression was significantly higher in both AML and ALL cells compared with PBMNC from healthy volunteers. In tumor-bearing mice, the plasma miR-92a level was significantly decreased in accordance with tumor growth, while tumor tissue was strongly positive for miR-92a.

**Conclusions:**

The miR-92a expression in leukemia cells could be a prognostic factor in ALL patients. The inverse correlation of miR-92a expression between cells and plasma and the cell to plasma ratio may be important to understanding the clinical and biological relevance of miR-92a in acute leukemia.

## Background

MicroRNAs (miRNAs) are a class of small noncoding RNAs of 19 to 25 nucleotides in size that are endogenously expressed in mammalian cells [1-3]. They regulate gene expression by repressing mRNA translation or cleaving target mRNA. As a new family of gene regulators, miRNAs are involved in the modulation of multiple cellular pathways, including cell proliferation, differentiation, and apoptosis. Recent evidence indicates that miRNAs can function as tumor suppressors and oncogenes, and they are therefore referred to as "oncomiRs" [4].

Evidence suggests that certain tumor-derived circulating miRNAs, such as miR-155, miR-21, miR-15b, miR-16, and miR-24, are detected in cell-free specimens, such as plasma and serum of cancer patients, regardless of the high RNAse activity in plasma; therefore, this may reflect tumor-derived miRNA [5,6]. In general, the level of miRNA in cell-free specimens is consistent with the levels found in tumor cells [5-7]. We previously identified miR-92a as a plasma biomarker in human leukemia [8]. We found that the miR-92a plasma expression level was significantly lower--approximately only 1/100th--in both acute myeloid leukemia (AML) and acute lymphoblastic leukemia (ALL) compared with normal controls. Since overexpression of the miR-17-92 cluster has been demonstrated in various neoplasias, including lymphoma and lung cancer [9-13], miR-92a is categorized as a possible oncomiR. The question has thus arisen regarding why the level of miR-92a is down-regulated in acute leukemia cells.

We therefore set out to determine miR-92a expression levels in acute leukemia cell lines and fresh acute leukemia cells. We used peripheral blood mononuclear cells (PBMNC) obtained from healthy volunteers as controls. We found high miR-92a expression in leukemia cell lines as well as in CD34-positive cells obtained from healthy volunteers. Although miR-92a expression in fresh leukemia cells was highly variable, miR-92a expression was significantly increased in a subset of ALL cells, and ALL patients with overexpressed miR-92a had poor outcomes. We then reanalyzed previously published data on plasma miR-92a expression [8] with respect to the expression in leukemia cells. We now show that the cell to plasma ratio of miR-92a expression is significantly higher for both AML and ALL compared with the ratio based on PBMNC from healthy volunteers. We also show that the regulation of miRNA is generally impaired in acute leukemia. Our results shed light on cell-free miRNA dynamics in cancer patients.

## Methods

### Patients and cells

In the current study, 91 patients aged 17-72 years with untreated, primary acute leukemia whose leukemia cells were more than 80% were included: 45 patients had ALL with pre-B phenotype and 46 had AML. CD34-positive cells from five healthy volunteers and PBMNC from 20 healthy volunteers were used as controls for cellular miR-92a expression. A study using plasma specimens was reported elsewhere [8]. This study was approved by the institutional review board of Tokyo Medical University. Written informed consent according to the Declaration of Helsinki was obtained from all patients prior to collection of the specimens. All 91 patients were treated according to the Japan Adult Leukemia Study Group protocols in the same institution. Human myeloid leukemia cell lines (HL-60, Kasumi-1, KOPM-K, U937, and K562), human lymphoid leukemia cell lines (BALL-2, OM9;22, HAL-01, NALM21, NALM24), and a human Burkitt lymphoma cell line (Raji) were also used in a part of this study.

### Quantitative reverse transcription polymerase chain reaction of mature miRNAs

CD34-positive cells of healthy volunteers were isolated using an Indirect CD34 MicroBead Kit and Magnetic Cell Sorting system (Miltenyi Biotec, Auburn, California), according to the manufacturer's instructions. Total RNA in cells was isolated using an miRNeasy Mini Kit (Qiagen, Germantown, Maryland), and RNA in plasma was extracted as reported previously [8]. MicroRNAs were quantified using TaqMan MicroRNA assays (Applied Biosystems, Foster City, California) with modifications. The microRNA specific stem-loop primers hsa-miR-92a (000431; Applied Biosystems), RNU6B (001093; Applied Biosystems), and hsa-miR-638 (Applied Biosystems) were used in the study. The expression of miR-92a was calculated using 2- ΔΔ*C^t ^*methods, and mean cycle threshold (*C^t^*) values for all miRNAs were quantified using sequence detection system software (SDS, version 1.02; Applied Biosystems). We used total RNA obtained from either plasma or PBMNC of healthy volunteers as controls, and the miR-92a expression levels in acute leukemia specimens were expressed as "ratios to controls".

### Antisense oligonucleotides and apoptosis assay

Antisense oligonucleotides and their respective scrambled control oligonucleotides were purchased from Applied Biosystems: an anti-miR-92a inhibitor (AM10916) and anti-miR negative control #1 (AM17011). To assess their effects, cells were plated to a density of 1 × 10^5 ^cells/well in 24-well dishes and then transfected with the oligonucleotides (50 nM) using a SuperFect Transfection Reagent (Qiagen) (day 1). The cells were then transfected again with oligonucleotides (50 nM) on day 5 and analyzed. The annexin V-binding capacities of the treated cells were examined using the annexin V-biotin apoptosis detection kit (Calbiochem, La Jolla, California), using an Agilent 2100 Bioanalyzer (Agilent, Wilmington, Delaware), as previously reported [14].

### The miR-92a expression in plasma and cells from tumor-bearing mice

For the in vivo assessment of miR-92a, 6-week-old female nude mice were injected with U937 cells. The miR-92a expression in plasma and tumor was measured on day 7 and day 18. Tumors were collected at the predetermined times and fixed in paraformaldehyde.

Paraffin-embedded tissues were sectioned and processed for gross histopathology by hematoxylin-eosin staining. Locked nucleic acid (LNA)-modified probes for miR-92a and negative control (miRCURY-LNA detection probe; Exiqon, Vedbaek, Denmark) were used to detect miR-92a expression in spleen and tumor, as reported previously [8].

### Statistical analysis

We used GraphPad Prism 5.0 software (GraphPad Software Inc., San Diego, California) for statistical analysis. A Mann-Whitney test was used to determine statistical significances between the control and test groups. One-way ANOVA was used to determine statistically significant relationships among more than three groups. *P*-values less than 0.05 were considered to indicate statistically significant differences.

## Results

### The miR-92a expression levels in immortalized leukemia cell lines and CD34-positive cells from healthy volunteers

**Figure 1:**
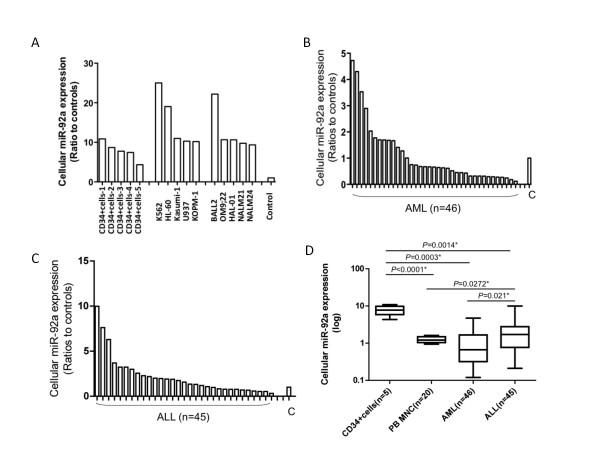
**MiR-92a expression in leukemia cells and in CD34-positive cells obtained from healthy volunteers**. A: Cellular miR-92a expression levels in CD34-positive cells obtained from normal individuals, AML cell lines, and ALL cell lines. C: Controls (PBMNC obtained from healthy volunteers.) B: MiR-92a expression in AML cells and control cells. C: MiR-92a expression in ALL cells and control cells. D: Comparison of miR-92a expression in acute leukemia cells as well as CD34-positive cells and PBMNC obtained from healthy volunteers.

We first examined miR-92a expression levels in leukemia cell lines as well as CD34-positive cells obtained from healthy volunteers (Figure 1A). Cellular miR-92a expression was expressed as ratios based on the average from normal PBMNC controls (ratios to controls in the Figure 1A), and we found miR-92a expression to be 5- to 10-fold higher in CD34-positive cells. Total RNA was extracted from a serum-free culture of cell lines to exclude contamination from bovine miRNA. Most of the immortalized cell lines showed more than 10-fold higher miR-92a expression compared with controls. In the five myeloid leukemia cell lines, miR-92a expression ranged from 10.2 to 25 (mean ± SD, 15.1 ± 6.665) and was significantly higher than in CD34-positive cells (mean ± SD, 7.814 ± 2.360; *P *= 0.0251). Similarly, in ALL cell lines, miR-92a expression ranged from 9.361 to 22.19 (mean ± SD, 12.52 ± 5.433) and tended to be higher than in CD34-positive cells (*P *= 0.0538).

These findings indicate that miR-92a expression may be linked to constant cell growth such as in immortalized leukemia cell lines and hemopoietic stem cells. In keeping with our previous observation using in situ hybridization of miR-92a in AML cells [8], leukemia cells possessed high levels of miR-92a expression.

### MiR-92a expression in fresh leukemia cells

Unlike leukemia cell lines, the miR-92a expression levels in fresh leukemia cells were generally low. In addition, these expression levels were highly variable compared with controls (mean ± SD, 1.257 ± 0.2261).

We found wide variation of miR-92a expression in fresh AML cells (Figure 1B): miR-92a expression ranged from 0.1199 to 4.724 (mean ± SD, 1.131 ± 1.112), and there was no significant difference in miR-92a expression between AML cells and PBMNC; however, miR-92a expression varied among FAB subtypes. The miR-92a expression in AML-M3 was significantly lower than in AML-M1 (*P *= 0.0325) (Additional file 1), indicating that miR-92a expression may be linked to cellular differentiation in AML cells.

Similar to expression in AML cells, miR-92a expression in ALL cells varied from 0.2109 to 9.987 (mean ± SD, 1.257 ± 0.2261) (Figure 1C) and was significantly lower than in CD34-positive control cells (*P *= 0.0014) (Figure 1D). There was no significant difference in miR-92a expression levels among cytogenetic ALL groups (Additional file 2).

In contrast to expression in AML cells, miR-92 expression in ALL cells was higher than in PBMNC (*P *= 0.0272), and miR-92a expression was significantly higher in ALL cells than in AML cells (*P *= 0.0021) (Figure 1D).

### Potential role of miR-92a in ALL cells

**Figure 2:**
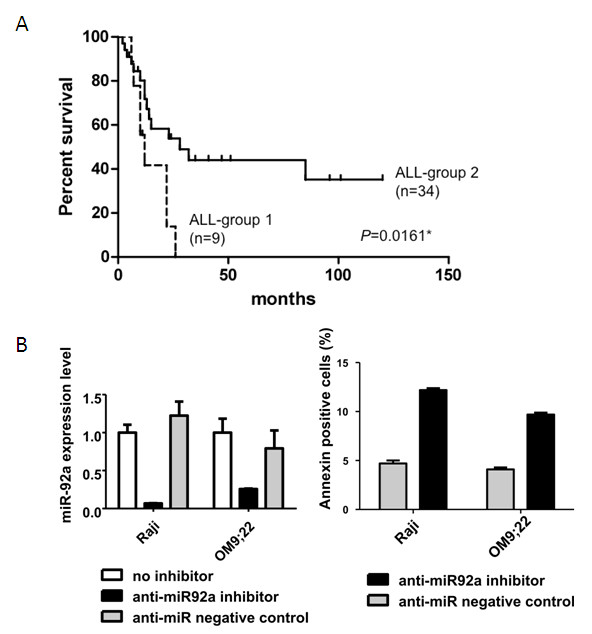
**Role of miR-92a expression in ALL cells**. A: ALL-group 1; miR-92a expression level is three times greater than that of normal controls (*n *= 9). ALL-group 2; miR-92a expression level is a third or less than normal controls (*n *= 34). Patients of ALL-group 1 had significantly shorter survival than ALL-group 2 patients (*P *= 0.0186). B: The left panel shows the efficacy of anti-sense oligonucleotides. MiR-92a expression level was decreased only when cells were treated with anti-miR-92a inhibitor. The right panel shows the anti-apoptotic effect of miR-92a in Raji and OM9;22 cells. Annexin-positive cells were more frequent in anti-miR-92a inhibitor-treated Raji and OM9;22 cells than in anti-miR negative control-treated cells.

On the basis of the results obtained from cellular miR-92a expression, miR-92a appears to play some role in ALL cells but not AML cells. We arbitrarily divided ALL patients into two groups; group 1 was composed of ALL patients whose miR-92a expression levels were greater than three times the average level of normal PBMNC controls (*n *= 9), and the remaining ALL patients, whose miR-92a expression levels were lower than threefold normal PBMNC controls, were assigned to group 2 (*n *= 34). Patients in group 1 had significantly shorter survival compared with those in group 2 (*P *= 0.0186) (Figure 2A). This suggests that cellular miR-92a levels may be linked to poor outcome in ALL patients.

To further elucidate the potential role of miR-92a in ALL cells, we used antisense oligonucleotides to examine the effects of reducing miR-92a expression in Raji and OM9;22 cells. Although the anti-miR-92a inhibitor did not inhibit growth in the cell lines (data not shown), we found an increase in annexin-positive cells in the anti-miR-92a inhibitor-treated Raji and OM9;22 cells compared with the anti-miR negative control-treated cells (Figure 2B). These findings suggest that miR-92a may act as oncomiRs in fresh ALL cells.

### Cell to plasma ratio of miR-92a expression is elevated in acute leukemia

**Figure 3:**
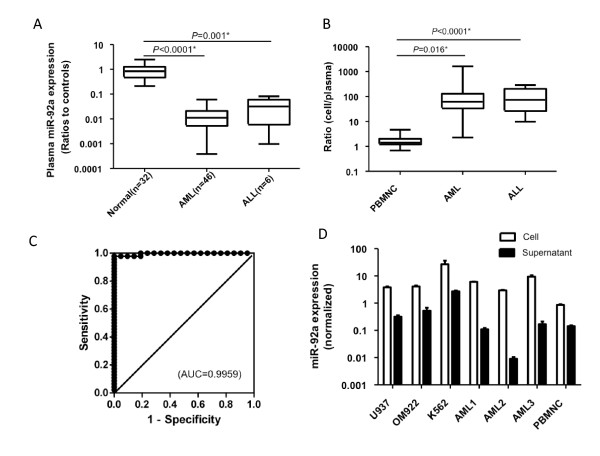
**Implication of plasma miR-92a**. A: Plasma miR-92a expression levels in acute leukemia patients and normal controls. B: Cell to plasma ratio of miR-92a expression. C: ROC curve of cell to plasma ratio in acute leukemia. D: The miR-92a expression in cells and supernatant. Cells were cultured under serum-free conditions for 24 hours, and then total RNA was extracted from both cells and supernatant. The expression levels of miR-92a in the supernatant are shown in closed boxes, and those in cells are show in open boxes.

We then reanalyzed previously published data on plasma miR-92a expression [8] with respect to its expression in leukemia cells. Since we could not detect U6B, which is commonly used as a internal standard for miRNA expression analysis in cells, we used miR-638 as a reference in each sample, as previously reported [8]. We could not find any obvious correlation between plasma miR-92a and cellular miR-92a in any case since plasma miR-92a levels were extremely low in most of the samples (Figure 3A). Eventually, we found that the cell to plasma ratio of miR-92a was significantly higher in both AML (*P *= 0.016) and ALL (*P *< 0.0001) cells compared with those in peripheral blood specimens obtained from healthy individuals (Figure 3B). Receiver operation characteristic (ROC) curve analysis clearly shows an area under the ROC curve (AUC) value of 0.9959 (Figure 3C). This may indicate that release or reuptake of miRNA by acute leukemia cells is different from that of peripheral blood cells obtained from healthy individuals.

To further elucidate the mechanism of release and reuptake of miR-92a by leukemia cells, we measured miR-92a expression in both cells and supernatant in vitro. Cells (5 × 10^5 ^cells/ml) were cultured in a serum-free medium for 24 hours, and miR-92a expression in both cells and supernatant was analyzed. We found various levels of miR-92a expression in the supernatant (Figure 3D). It is likely that the in vitro dynamics of miR-92a are not reflected in living cells.

### Low plasma miR-92a expression in tumor-bearing mice

**Figure 4:**
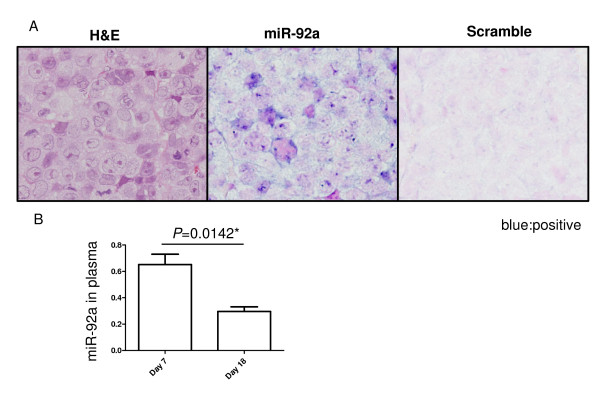
**The miR-92a expression in tumor-bearing mouse**. A: Tumor tissue in nude mouse. Tumor tissue was stained with hematoxylin and eosin (HE; left). In situ hybridization was performed using LNA probes for miR-92a. The tumor tissue was positive for miR-92a. Blue signals represent positive results for miR-92a. In situ hybridization was performed using LNA probes for the scramble probe. The tumor tissue was negative for the scramble probe (right). B: Plasma miR-92a levels were significantly decreased in accordance with tumor growth (*P *= 0.0142).

To determine the expression levels of miR-92a in xenotransplanted tumor tissues, we performed in situ hybridization using LNA-modified probes labeled with digoxigenin. We found that miR-92a was strongly expressed in tumors, while tumor tissue was negative for the scramble probe (negative controls) (Figure 4A). Although we could not rule out the possibility that mouse miR-92a affects the level of miR-92a in mice plasma, plasma miR-92a levels were significantly decreased in accordance with tumor growth (*P *= 0.0142)(Figure 4B).

## Discussion

This is the first report to circumstantiate the dysregulation of miRNA in patients with acute leukemia by analyzing miR-92a expression in both cells and plasma. First, miR-92a expression was increased in a subset of ALL cells, and the expression pattern may be linked to clinical behavior in ALL patients. Second, the cell to plasma ratio was low in both AML and ALL, indicating the possibility that leukemia cells retain miR-92a.

In AML, several studies have described the association between cellular miRNA expression and chromosomal abnormalities [15-17]. For example, up-regulation of miR-127/125b/154/323/368/370/381 is associated with leukemia cells with *t*(15;17), and up-regulation of miR-126* is associated with those with *t*(8;21). It is likely that certain chromosomal abnormalities or genetic changes, such as FLT3-ITD and NPM1 mutations, may be more important in defining the biological properties of AML cells. Indeed, we could not determine the clinical relevance of miR-92a when analyzing only a cell fraction of AML.

In contrast, we found cellular miR-92a appears to play some role in a subset of ALL patients. This result was not surprising since the miR-17-92 cluster is known to be related to lymphoid development as well as lymphomagenesis [13]. However, our results highlight the biological and clinical properties of miR-92a in ALL cells in that patients with higher expression of miR-92a had significantly shorter survival. Although the knock-down of miR-92a did not show growth inhibition, an increase in apoptotic cells may indicate that miR-92a possibly inhibits apoptosis in a subset of ALL cells. Recent reports by Kotani et al. [18] demonstrated that the expression of two tumor-suppressive miRNAs, miR-128b and miR-221, was down-regulated in *MLL*-rearranged ALL relative to other types of ALL and was related to glucocorticoid resistance. Zhang et al. [19] also reported that miR-34a, miR-128a, miR-128b, and miR-146a are associated with prednisolone response in pediatric ALL. Kaddar et al. [7] have shown that miR-16 acts as an oncomiR in childhood ALL and is thereby related to poor outcome. In accordance with the current study, there may be several prognostic parameter-associated miRNAs in ALL, although precisely which target genes or pathways are linked to which miRNA effects is still unclear.

Several lines of evidence suggest that miRNA in cells as well as circulating tumor-derived miRNA (i.e., in saliva, urine, and plasma) plays an important role in cancer development and progression [5,6,20-23]. Unlike miRNA in cancer cells, the biological relevance of circulating tumor-derived miRNA has not been fully elucidated. In general, the level of miRNA in cells is consistent with the levels in cell-free specimens such as plasma [7]. For example, Mitchell et al. [6] found that plasma miR-141 is overexpressed in prostate cancer patients. Also, Lawrie et al. [5] found that patients with diffuse large B-cell lymphomas had high serum levels of miR-21, which was associated with increased relapse-free survival. More recently, plasma miR-29a and miR-92a have been found to be significantly overexpressed in colorectal cancers compared with normal controls [21]. These findings suggest that circulating oncomiR expression levels are generally high in cancer patients; thereby, the cell to plasma ratio of miRNA might be constant.

In this study, the cell to plasma ratio is remarkably elevated in acute leukemia cells compared with the ratio based on PBMNC obtained from healthy volunteers. Our results showed a discrepancy between oncomiR expression in cells and plasma. Park et al. [24] demonstrated that miR-200a, which is commonly overexpressed in oral squamous cell carcinoma cell lines, was present in significantly lower levels in the saliva of patients with oral squamous cell carcinoma than in the saliva of control subjects [23]. The most plausible explanation for this discrepancy is that cancer-specific miRNAs undergo a more rapid degradation and/or have a shorter half-life during the cell death process, similar to the degradation of regulatory mRNAs [25]. It is also possible that release of miR-92a is impaired in acute leukemia cells. Zernecke et al. [26] demonstrated that extracellular exosomal miRNAs were transferred into other cells such as endothelial cells. More recently, Kosaka et al. [27] also demonstrated secretary mechanisms and intercellular transfer of miRNA in living cells. Taking the information as a whole, we could not rule out the possibility that the miR-92a in plasma may transfer to other cells in acute leukemia. For this reason, the cell to plasma ratio may be important in understanding the biological relevance of miR-92a in acute leukemia.

## Conclusions

We have shown a possible role of miR-92a in ALL cells. The quantification of miR-92a in leukemia cells and plasma may be used for monitoring leukemia in both ALL and AML patients. Although questions still remain about the underlying mechanism of miR-92a dynamics in patients with acute leukemia, our results highlight the impact of the cell to plasma ratio of miR-92a in patients with acute leukemia. Obviously, the signal network mediated by miR-92a needs to be clarified. Our results anticipate a possible role of circulating miRNA in cancer patients.

## Abbreviations

ALL: acute lymphoblastic leukemia; AML: acute myeloid leukemia; LNA: locked nucleic acid; miRNA: microRNA; PBMNC: peripheral blood mononuclear cells; RT-PCR: reverse transcription polymerase chain reaction; FAB: The French-American-British (FAB) classification; M1: acute myeloblastic leukemia, without maturation; M2: acute myeloblastic leukemia, with granulocytic maturation; M3: promyelocytic, or acute promyelocytic leukemia.

## Competing interests

The authors declare that they have no competing interests.

## Authors' contributions

JHO participated in the design and interpretation of the analysis, statistical analysis, and the writing of the article. TU planned and coordinated the research. CK and RH collected miRNA samples from patients and performed RT-PCR analysis. MT provided quality control data. MK helped in the interpretation of results. KO helped to write the article. All authors read and approved the final manuscript.

## Supplementary Material

Additional file 1 **MiR-92a expression levels and FAB subtypes in AML**. MiR-92a expression levels in M1 were significantly higher than those in M2 (*P *= 0.0033) and M3 (*P *= 0.0325). Click here for file

Additional file 2 **There was no significant difference in miR-92a expression levels among ALL cytogenetic groups**. Click here for file
