# Supplementary figures and images for: Impact on cell to plasma ratio of miR-92a in patients with acute leukemia: in vivo assessment of cell to plasma ratio of miR-92a
Source: BMC Res Notes. 2010 Dec 24;3:347. doi: 10.1186/1756-0500-3-347 (PMC3022817; doi:10.1186/1756-0500-3-347)

## Slide 1
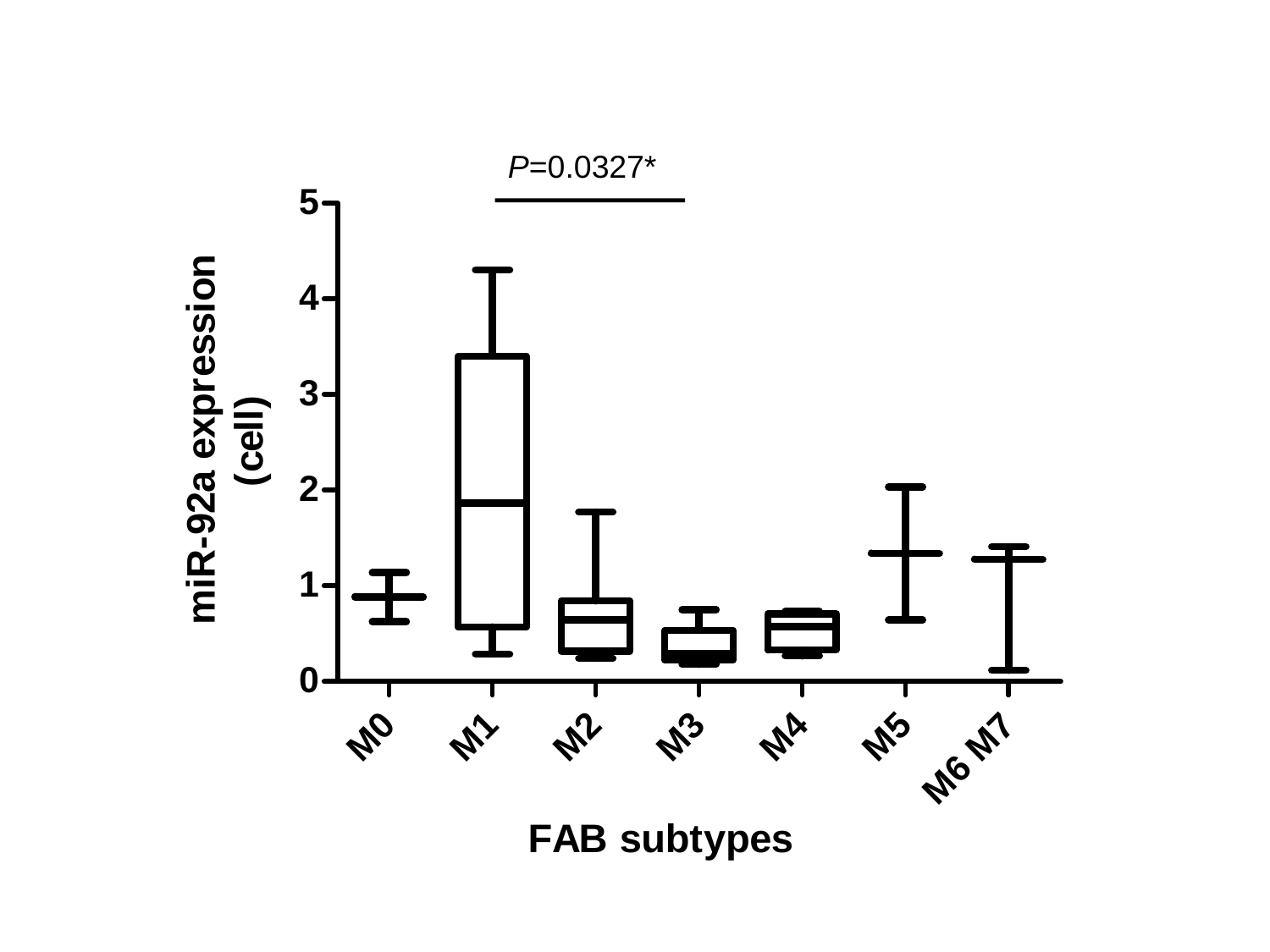

P=0.0327*

Supplement: Additional file 1 — MiR-92a expression levels and FAB subtypes in AML. MiR-92a expression levels in M1 were significantly higher than those in M2 (P = 0.0033) and M3 (P = 0.0325). [file 1756-0500-3-347-S1.PPT]

## Slide 1
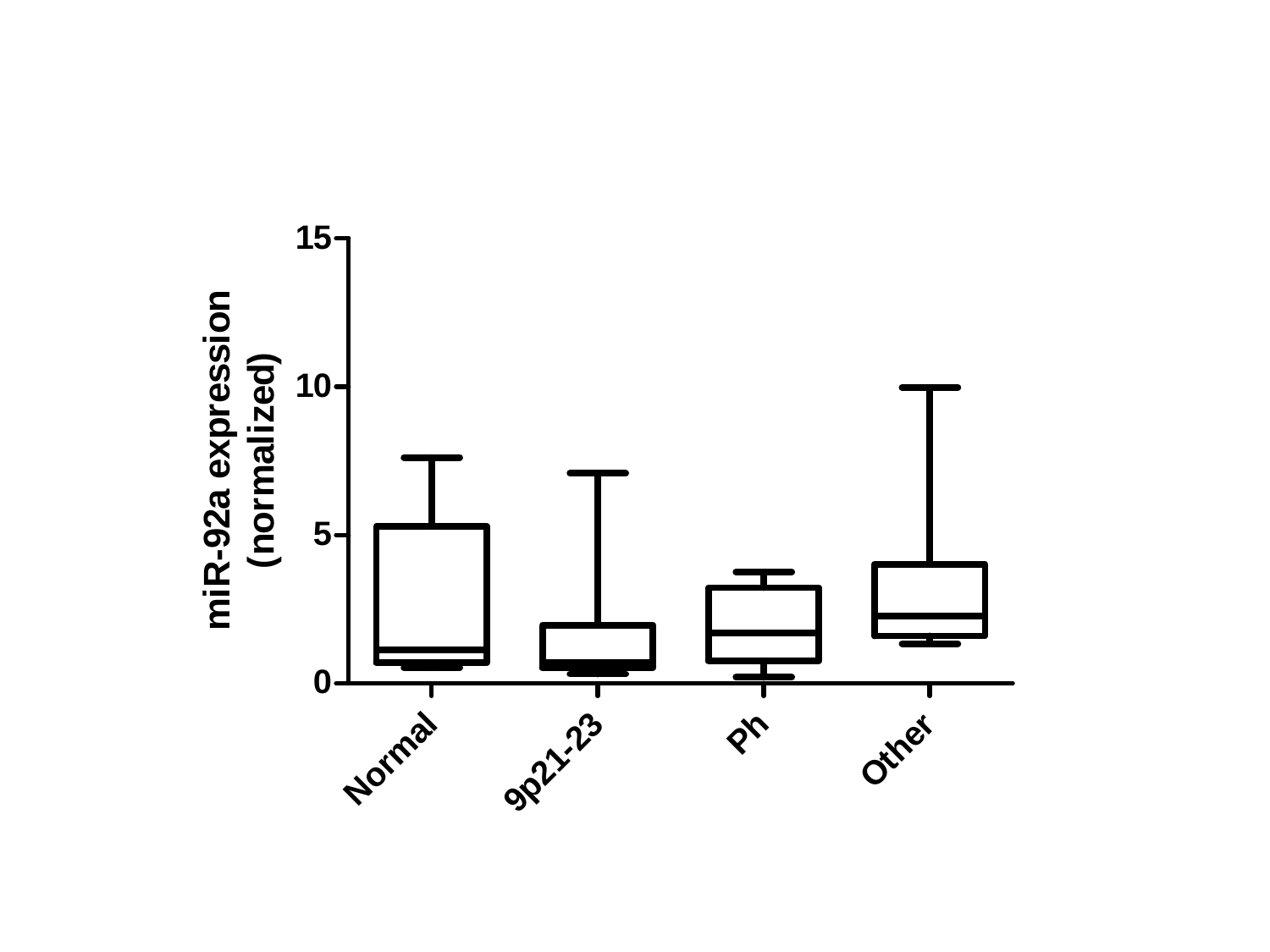

Supplement: Additional file 2 — There was no significant difference in miR-92a expression levels among ALL cytogenetic groups. [file 1756-0500-3-347-S2.PPT]
